# Occurrence and Molecular Characterization of Multidrug-Resistant Vegetable-Borne *Listeria monocytogenes* Isolates

**DOI:** 10.3390/antibiotics11101353

**Published:** 2022-10-04

**Authors:** Zizipho Ntshanka, Temitope C. Ekundayo, Erika M. du Plessis, Lise Korsten, Anthony I. Okoh

**Affiliations:** 1 SAMRC Microbial Water Quality Monitoring Centre, University of Fort Hare, Alice 5700, South Africa; 2 Applied and Environmental Microbiology Research Group, Department of Biochemistry and Microbiology, University of Fort Hare, Alice 5700, South Africa; 3 Department of Biological Sciences, University of Medical Sciences, Ondo City PMB 536, Nigeria; 4 Department of Plant and Soil Sciences, Faculty of Natural and Agricultural Sciences, University of Pretoria, Pretoria 0002, South Africa; 5 Department of Environmental Health Sciences, College of Health Sciences, University of Sharjah, Sharjah P.O. Box 27272, United Arab Emirates

**Keywords:** vegetable, antibiotic resistance gene, multidrug-resistant, multiple antibiotic resistance (MAR) index, risk, public health

## Abstract

Fresh vegetables play a significant role in the human diet. However, ready-to-eat (RTE) vegetables have been associated with increasing foodborne outbreaks including *L. monocytogenes*, which is a common human pathogen associated with foodborne infections resulting in listeriosis. This study aims to assess the resistance of vegetable-borne *L. monocytogenes* to antibiotics. *L. monocytogenes* was isolated and molecularly characterized using polymerase chain reaction (PCR) from 17 RTE vegetable samples. The confirmed *L. monocytogenes* was further assessed for phenotypic and genotypic antibiotic resistance using the disc diffusion test and PCR primers targeting six antibiotic classes and thirty-one related antibiotic resistance genes (ARGs), respectively. The results revealed that *Listeria* counts ranged from 1.60 to 3.44 log_10_ CFU/g in the samples. The isolates exhibited high resistance against penicillin G, erythromycin, vancomycin, tetracycline, trimethoprim-sulfamethoxazole, and nitrofurantoin among the 108 isolates tested. A total of 71 multiple antibiotic resistance (MAR) phenotypes were observed in the isolates, which ranged from resistance to 3 to 13 antibiotics. The MAR index was ˃0.2 in 97% of the isolates. Some of the highly detected ARG subtypes included *SulI* (100%), TEM (76.9%), *tetA* (59%), and *tetM* (54.7%). The findings show a high occurrence of multidrug-resistant *L. monocytogenes* and clinical ARGs in fresh vegetables, which constitutes an immediate danger for the health security of the public.

## 1. Introduction

*Listeria monocytogenes* is a common Gram-positive facultative anaerobic bacillus that is recognised as one of the most important foodborne pathogens by the World Health Organisation [1]. Based on latest classification, the genus *Listeria* is comprised of 20 major species [2] and is organised into two groups centred on their relationship with *L. monocytogenes* [3]—a known pathogen of the human foodborne infection, listeriosis [4]. *L. monocytogenes* is an invasive pathogen that can infect human and animals. It can cause severe listeriosis, leading to meningoencephalitis, sepsis, and foetal infection or miscarriage in pregnant women, with a mortality rate of 20–40% [1]. Antibiotic therapies usually employed in the treatment of listeriosis include penicillin, ampicillin, gentamicin, rifampicin, chloramphenicol, erythromycin, tetracycline, or trimethoprim with sulphamethoxazole in combination or alone [5,6]. However, most *L. monocytogenes* strains possess natural resistance to currently used fluoroquinolones and cephalosporins—particularly those of the third and fourth generations [7].

*L. monocytogenes* occurs naturally in agricultural settings such as irrigation source water, soil, and fertilizers used on farms and in rotting plant matter, making its presence in vegetables a continuous risk [6]. To impede the multiplication of microbes and to ensure adequate preservation, some vegetables are stored and transported at low temperatures [6]. However, these conditions favour the growth of some microbial pathogens, such as *L. monocytogenes*, a psychotropic microorganism that is highly significant to public health [6,8,9]. *L. monocytogenes* has the ability to grow over varied temperatures, including freezing temperatures, and even in pH levels as low as 4.4, high salt content, low moisture content, and the absence of oxygen [10,11,12]. Hence, it is prevalent in samples from clinics and foods [10,11,12].

Recent reports show that there is an increase in contamination by and the occurrence of *L. monocytogenes* in fresh produce [13] (https://www.foodsafetynews.com/2022/06/no-details-on-new-outbreak-of-listeria-infections-fda-working-on-other-outbreaks/, accessed on 6 June 2022). This organism has been isolated from eateries or market produce such as carrot, cabbage, cucumber, and salad vegetable produce [13]. Outbreaks of *L. monocytogenes* infections linked to fresh produce have been reported all around the world [14]. Recently, an outbreak of listeriosis reported in South Africa involved 1060 cases, with 216 presumed dead (NICD, 2018). Information on the incidence of *L. monocytogenes* in fresh vegetables in the Sarah Baartman District Municipality in the Eastern Cape Province, South Africa is rare; hence, this study aimed at assessing the occurrence of multidrug-resistant *L. monocytogenes* in fresh vegetables from the Sarah Baartman District Municipality in the Eastern Cape Province, South Africa. Furthermore, it aimed to molecularly characterise the resistance genes involved in the multidrug-resistant vegetable-borne *L. monocytogenes.*

## 2. Results

### 2.1. Prevalence of Listeria Species in Vegetables

**Figure 1:**
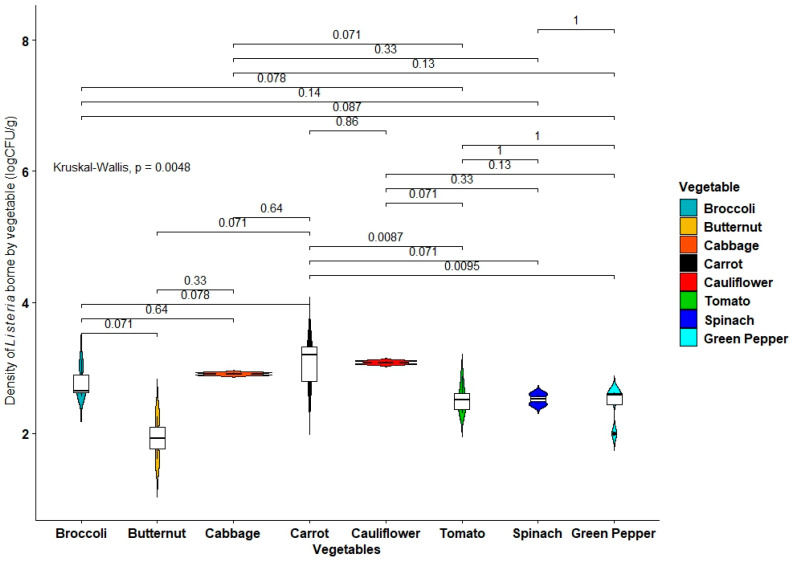
Distribution of presumptive *Listeria* species in vegetable samples.

The distributions (in log_10_ CFU/g) of *Listeria* spp. in the different vegetable samples studied are shown in Figure 1. The counts ranged from 1.60 to 3.44 log_10_ CFU/g, with the highest observed in carrots. The overall average of *Listeria* counts in the samples was 2.70 ± 0.45 log_10_ CFU/g. Generally, *Listeria* spp. counts were significantly different among the vegetables (Kruska–Wallis, *p* = 0.0084), with higher values in cabbages, cauliflowers, and carrots compared with the overall average.

### 2.2. Incidence of Vegetable-Borne Listeria monocytogenes

**Figure 2:**
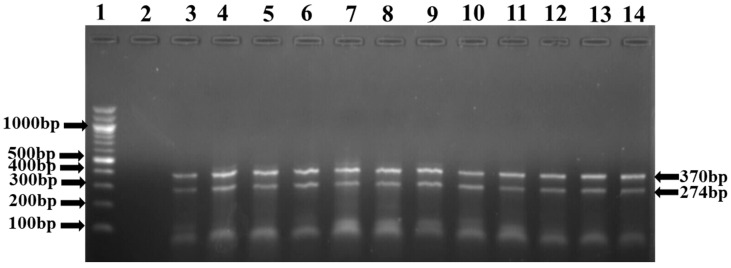
PCR products of the amplification *prs* (370 bp) and *prfA* (274 bp) genes for the detection of *listeria* genus and *Listeria monocytogenes*, respectively. Lane 1: Molecular weight Marker (100 bp); Lane 2: Negative control, Lane 3–14: *Listeria* species.

A total of 189 presumptive *Listeria* isolates were screened for the presence of the *prs* and *prfA* genes for the identification of *Listeria* genus and *L. monocytogenes*, respectively, using a duplex PCR assay. While 59% (112/189) of the presumptive isolates were confirmed as *Listeria* spp., 57% (108/189) were confirmed as *L.* *monocytogenes* (Figure 2).

### 2.3. Antibiotic Susceptibility Patterns of L. monocytogenes

**Figure 3:**
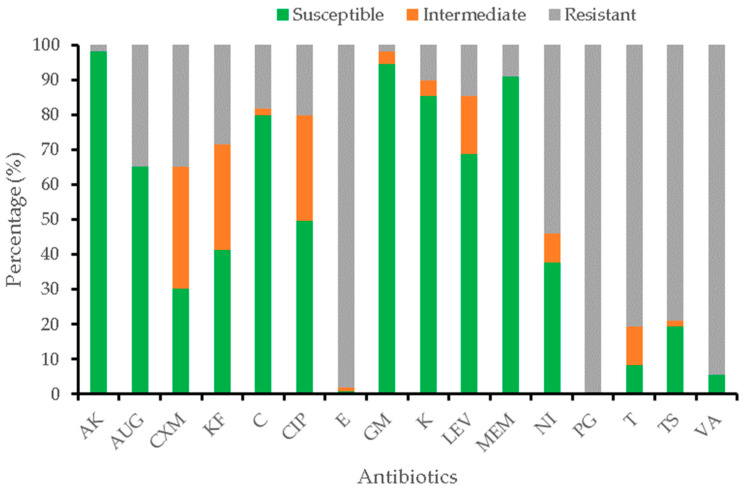
Antibiogram profile of *L. monocytogenes* isolates against selected antibiotics. AK = Amikacin; AUG = Amoxicillin-clavulanic acid; CXM = Cefuroxime; KF = Cephalotin; C = Chloramphenicol; CIP = Ciprofloxacin; E = Erythromycin; GM = Gentamicin; K = Kanamycin; LEV = Levofloxacin; MEM = Meropenem; NI = Nitrofurantoin; PG = Penicillin G; T = Tetracycline; TS = Trimethoprim-Sulfamethoxazole; VA = Vancomycin.

One hundred and eight positive *L. monocytogenes* isolates were assessed for their phenotypic antibiotic resistance patterns against 16 different antibiotics (Figure 3). All isolates showed resistance against penicillin G. High levels of resistance were equally observed against erythromycin, vancomycin, tetracycline, and trimethoprim-sulfamethoxazole. However, the isolates were found to be highly sensitive to amikacin, gentamicin, meropenem, kanamycin, chloramphenicol, levofloxacin, and amoxicillin-clavulanic acid.

### 2.4. Multiple Antibiotic Resistance Phenotypes (MARP) of L. monocytogenes

The MAR phenotype patterns and the multiple antibiotic resistance index (MARI) of all *L. monocytogenes* isolates are given in Table 1. The isolates exhibited a total of 71 patterns of MARPs ranging from resistance to 3 to 13 antibiotics, with a high number of single appearances. Ninety-seven percent of the isolates had MARI values that were greater than the 0.2 threshold value, which is the recommended benchmark for MARI as set by Krumperman [15].

### 2.5. Prevalence of Clinical Antimicrobial Resistance Gene (ARGs) Subtypes

**Figure 4:**
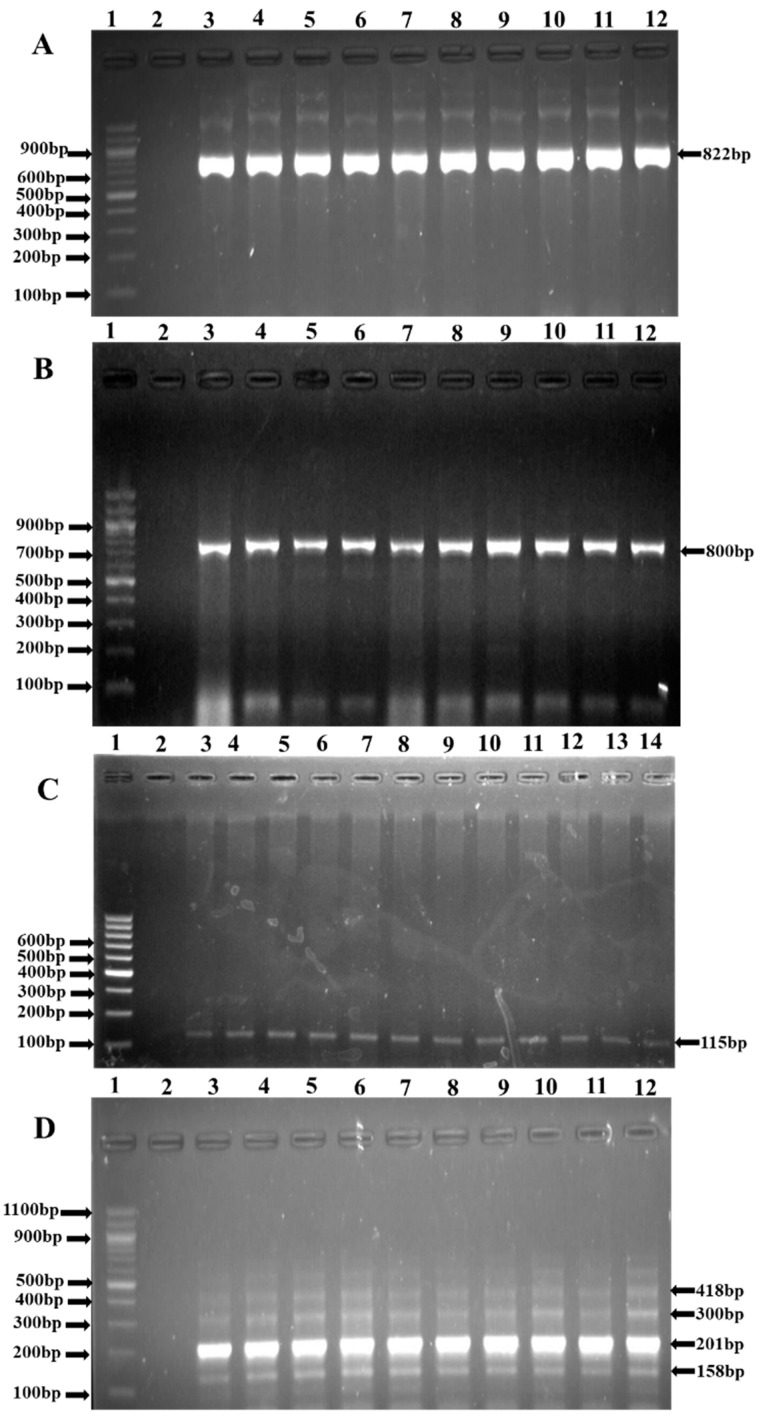
Representative PCR products of some of the targeted resistance genes. (**A**): Gel picture showing *sul*I resistance gene (822 bp). Lane 1: Molecular weight Marker (100 bp); Lane 2: Negative control; Lanes 3–12: positive isolates. (**B**): Gel picture showing *bla*_TEM_ resistance gene (800 bp). Lane 1: Molecular weight Marker (100 bp); Lane 2: Negative control; Lanes 3–12: positive isolates. (**C**): Gel picture showing *cml*A1 resistance gene (115 bp). Lane 1: Molecular weight Marker (100 bp); Lane 2: Negative control; Lanes 3–14: positive isolates. (**D**) Multiplex PCR products for the amplification of *tet*A (201 bp), *tet*C (418 bp), *tet*D (300 bp), and *tet*M (158 bp). Lane 1: Molecular weight Marker (100 bp); Lane 2: Negative control; Lanes 3–12: positive isolate.

Details on the prevalence of resistance genes detected in *L. monocytogenes* isolates are given in Table 2. The resistance genes detected included some relevant ARGs and Extended-Spectrum β-Lactamase (ESBL) genes such as *tet*A, *tet*C, *tet*D, *tet*K, *tet*M, *aphA2*, *aadA*, *BlaTEM*, *amp*C, *TEM*, *PER, FOX*, *DHA*, *CIT*, *cmlA1*, *catII*, and *SulI* (Figure 4).

## 3. Discussion

Although only 17 vegetable samples were investigated for *Listeria* spp. and *L. monocytogenes* in the study, multiple isolates were picked from single samples to explore the variability among isolates that could be cultured from the same vegetable type—which also allowed for the investigation of diversity in susceptibility to antibiotics that may be present among the isolates.

The *Listeria* species detected and identified from the vegetable samples ranged from 3.04–4.38 log_10_ CFU/g. This suggests that the vegetables could have been contaminated on farms via irrigation water and other farm practices. Additionally, the count in the samples exceeded the 2 log_10_ CFU/g (100 CFU/g) maximum limit of *L. monocytogenes* for low-risk foods, as well as the “zero-tolerance” limit for ready-to-eat (RTE) foods. Thus, the vegetables are unsafe for raw consumption without thorough preparation and must be properly cooked before consumption. Public Health England (2014) reported that food samples containing ≥100 CFU/g (2 log_10_ CFU/g) of *Listeria* species are considered of unsatisfactory microbial quality and should be further investigated. Therefore, the vegetable samples studied pose a threat to consumers’ health and make them prone to the risk of contracting listeriosis from consumption of contaminated vegetables. However, the range of *Listeria* counts from the vegetables studied was lower compared to the 2.98–5.32 log_10_ CFU/g) previously reported by Bilung and colleagues [5] in vegetables.

The *Listeria* genus and *L. monocytogenes* contamination in the vegetables studied was 59% and 57%, respectively. The incidence of *Listeria* species from vegetables in this study was higher than that reported by Bilung and colleagues (6.7–8%) [5], Goni and colleagues (21%) [16], and Sangeetha and Shubha (1.81%) [17]. However, the *Listeria* incidence was lower than the *Listeria* incidence (69.2%) reported by Onyilokwu and colleagues in vegetables [18]. The occurrence of *L. monocytogenes* known to cause listeriosis in both humans and animals [19], at 57% among the vegetable-borne *Listeria isolates*, could be linked to pollution of irrigation source water with effluents from poultry and other husbandries—as seen upstream of the farm where some of the vegetables were collected. Similarly, irrigation water pollution or organic fertilizer usage might be prevalent in the farms that supply supermarkets and street vendors with vegetables in the catchment. *L. monocytogenes* contamination of vegetables has been found to occur through irrigation water used in fresh produce cultivation via surface contamination and internalization through the roots and subsequent survival in crops [20]. Raw vegetables are generally prone to *L. monocytogenes* contamination [21,22]. The 57% incidence of *L. monocytogenes* among the vegetable-borne isolates in this study is higher compared with the *L. monocytogenes* incidence of 4.18% and 13.6% reported by Moreno et al. [23] and Cetinkaya et al. [24], respectively, in vegetables.

The 108 *L. monocytogenes* profiled for antibiotic susceptibility showed resistance against penicillin G (100%), followed by erythromycin (98.2%), vancomycin (94.5%), tetracycline (80.7%), trimethoprim-sulfamethoxazole (78.9%), and nitrofurantoin (54.1%). This is an indication that the isolates originated from sources where high level of antibiotics have been used. Additionally, *L. monocytogenes* is known and capable of acquiring resistance against erythromycin and tetracycline from lactic acid bacteria [6], which are common in decaying vegetables and plant materials on farms as well as in food materials. The low resistance of the *L. monocytogenes* against gentamicin, one of the antibiotics of choice for the treatment of listeriosis in South Africa, ensures hope for the treatment of its infections in the locality. The gentamicin susceptibility profiles of the isolates are comparable to those reported by Bilung et al. [5] and Li et al. [25] in vegetables. The present results demonstrate a decrease in the efficacy of some of the antibiotics against *L. monocytogenes*. Thus, the vegetables represent a potential source of multidrug-resistant *L. monocytogenes* infections in the locality. Kuan et al. [26] observed 100% resistance to penicillin G—which is line with the findings of this work—and also found gentamicin (91.4%) to be effective in restraining the growth of *L. monocytogenes*. The reduced susceptibility of penicillin G as a first line drug may be caused by the indiscriminate use or misuse of this antibiotic, as reported by Kuan et al. [26]. The high level of resistance to penicillin G and vancomycin is very concerning as these antibiotics are used in the treatment of listeriosis during pregnancy and in treating listerial meningitis, respectively [27]. It is also worrisome that high resistance was recorded against trimethoprim-sulfamethoxazole as this antibiotic, together with vancomycin, can be used as an alternative therapy for patients allergic to penicillin [28].

The MAR phenotypes compiled for *L. monocytogenes* indicated a high degree of multiple antibiotic resistance, with a range of resistance to 3 to 13 antibiotics. The most prevalent MARPs were observed in MARP 6. E-PG-T-TS-VA (*n* = 6, 5.5%), E-NI-PG-T-TS-VA (*n* = 6, 5.5%), AUG-CXM-KF-C-E-K-NI-PG-T-TS-VA (*n* = 6, 5.5%), and AUG-E-NI-PG-T-TS-VA (*n* = 5, 4.6%) were the most predominant MARPs. This is indicatory of the possible failure of combinational therapy with these antibiotics should human or animal infections occur through them. The health risk associated with the spread of antibiotic resistance in an environment is estimated using MARI [29,30], and the MARI values obtained in this study signify an overuse or misuse of antibiotics in the environment from which the samples were collected. A MARI value of 0.2 (arbitrary) was used to differentiate between a low- and high-health risk. A value that is greater than 0.2 suggests that the resistant *L. monocytogenes* isolates originated from an environment where there is high contamination or overuse of antibiotics [29,30]. The MARI values of most of the vegetable-borne *L. monocytogenes* in this study was ≥0.2. This suggests that the *L. monocytogenes* originated from farms using irrigation water polluted with high levels of antimicrobial substances, antibiotic residue-laden effluents from livestock farming, or the application of organic manures with high loads of antimicrobial residues and antibiotic-resistant bacteria. This could further increase the spread of multidrug-resistant *Listeria* isolates to humans via the consumption of contaminated vegetables. This is similar to the *L. monocytogenes* MARPs reported from fruits and vegetables by Kayode and Okoh (2022) [31].

This study also investigated the prevalence of antibiotic resistance genes in *L. monocytogenes* isolates. A total of 14/25 antibiotic resistance genes were detected across six different antibiotic classes. Some of the isolates exhibited one or more antibiotic resistance genes that may act as a pool of resistance genes for other commensal and pathogenic bacteria in vegetable farm environments [32]. *SulI* (100%) was the only detected gene conferring resistance to sulfanomides. Sulfanomide-resistance genes signify that the isolates originated from animal sources. The most prevalent were genes conferring resistance to tetracyclines, including *tet*A (59%), *tet*M (54.7%), *tet*C (43%), and *tet*D (43%; Table 2). In beta-lactams, *bla*_TEM_ (76.9%) was among the other genes that were detected, including aminoglycosides *aph*A2 (41.7%), *aad*A (33.3%), and several other genes conferring resistance against phenicols, cephems, and aminoglycosides. However, high resistance against sulfamethoxazole (80.58%), amoxicillin (58.25%), and erythromycin (49.52%) was observed. About 85.44% of Lm isolates showed multidrug-resistant phenotypes against the tested antibiotics [31].

Conversely, some of the *L. monocytogenes* isolates displayed phenotypic resistance to multiple antibiotics but did not contain antibiotic resistance genes. *L. monocytogenes* isolates may have acquired genes for antibiotic resistance through antibiotic selection pressure or through various gene transfer mechanisms from other bacteria in the farm area [32]. Studies have shown the conjugative transfer of antibiotic resistance, i.e., the acquisition of enterococcal and streptococcal plasmids into the genus *Listeria* and subsequent transfer of these plasmids within the genus, including transmission to *Listeria monocytogenes* [33]. According to Srinivasan et al. [32], the expression of different genes of resistance to antibiotics does not always correlate with the phenotypic antibiotic resistance of foodborne pathogens.

## 4. Materials and Methods

This study was conducted in the Sarah Baartman District Municipality (SBDM), the largest district municipality in the Eastern Cape, with the geographical coordinates: 33°57′00” S; 25°36′00” E. Samples were collected from one farm, two supermarkets, and one street vendor. The selected farm, which is located near a river, supplies fresh vegetables to a number of Supermarkets across the SBDM, while the supermarkets are the most commonly used by the majority of residents from Grahamstown and its surrounds. The samples were aseptically collected in September 2018 using sterile stomacher bags and were transported to the laboratory, where they were analysed within 6 h of collection. Ethical clearance for the research was obtained from the University of Fort Hare Research Ethics board under the reference number: OKO011SNTS01.

For enrichment, 0.1 mL of each homogenized sample was cultured in 10 mL tryptic soy broth (TSB) and incubated at 37 °C for 18–24 h (Merck, Johannesburg, South Africa). For secondary enrichment, a 0.1 mL aliquot was transferred from the TSB suspension into 10 mL Fraser broth (Oxoid Ltd., Bsingstoke, UK) and incubated at 37 °C for 48 h. A loopful of the enriched culture was streaked onto chromogenic *Listeria* agar (LCA; Oxoid Ltd., Basingstoke, UK), and supplemented using an OCLA (ISO) selective supplement with an OCLA (ISO) differential supplement, following the standards defined by Ottaviani and Agosti (ALOA) in ISO 11290–1:1997, and incubated at 37 °C for 24 h. After incubation, the petri dishes were observed for typical *Listeria* species colonies (blue/green colonies with a sunken centre), which were picked and subsequently purified onto nutrient agar (NA; Oxoid Ltd., Basingstoke, UK) and incubated at 37 °C for 24 h. Pure distinct colonies were further inoculated in sterile nutrient broth (NB) and incubated at 37 °C for 18–24 h. The overnight culture was stored at –80 °C in a 25% glycerol stock until further analysis.

DNA was extracted from reactivated bacterial cells using the boiling method [34]. The bacterial cells were reactivated by inoculation into nutrient broth (NB), followed by incubation at 37 °C for 18–24 h. A loopful of the NB culture was further streaked onto NA and incubated at 37 °C for 24 h. Single distinct bacterial colonies were homogenised in 200 µL sterile nuclease-free water contained in an Eppendorf tube and boiled using a heating block (MS2a Dri-Block DB.2A Techne, Bibby Scientific LTD, Staffordshire, UK) at 100 °C for 10 min. Homogenates were further centrifuged at 13,500 rpm for 10 min to separate the liquid from the cell debris. The supernatant was kept at 4 °C and later used as a DNA template in PCR. Primers targeting the *prs* (F: 5′-GCT GAA GAG ATT GCG AAA GAA G-3′; R: 5′-CAA AGA AAC CTT GGA TTT GCG G-3′) and *prf*A (F: 5′-GAT ACA GAA ACA TCG GTT GGC-3′; R: 5′-GTG TAA TCT TGA TGC CAT CAG-3′) genes for *Listeria* genus and *L. monocytogenes*, respectively, were used for the amplification of DNA in PCR. PCR was performed in a 25 μL reaction volume consisting of 12.5 μL master mix (Quick-Load BioLabs), 4.5 μL nuclease-free water, 1 μL forward primer, 1 μL reverse primers, 5 μL DNA, 0.5 μL MgCl_2_, and buffer. The cycle condition included initial denaturation (94 °C, 5 min), followed by 33 cycles of denaturation (95 °C, 45 s), annealing (60 °C, 30 s), extension (72 °C, 1 min), and a single final extension (72 °C, 5 min). PCR products (5 µL) were electrophoresed in 1.5% agarose gel, stained with ethidium bromide solution, and visualised under a UV illuminator (Alliance 4.7 XD-79.WL/26MX, Paris, France).

The antibiotic resistance profiles of the confirmed isolates were assessed using the sixteen antibiotic panels recommended for the clinical treatment of *L. monocytogenes* infections by the Clinical and Laboratory Standards Institute [35] via Kirby–Bauer disc diffusion tests. The test antibiotics (Davies Diagnostics (Pty) Limited, Randburg, South Africa) included Meropenem (10 μg), Cefuroxime (30 μg), Gentamycin (10 μg), Erythromycin (15 μg), Vancomycin (30 μg) Cephalotin (30 μg), Ciprofloxacin (5 μg), Kanamycin (30 μg), Levofloxacin (5 μg), Chloramphenicol (30 μg), Trimethoprim-Sulphamethoxazole (25 μg), Nitrofurantoin (200 μg), Amikacin (30 μg), Tetracycline (30 μg), Amoxicillin clavulanic acid (300 μg), and Penicillin-G (10 μg). From a 24 h culture of *L. monocytogenes* isolates on nutrient agar, a single colony was picked and inoculated into 5 mL normal saline and vortexed. The turbidity of the suspension was adjusted to 0.5 McFarland standard (corresponding to 1.5 × 10^8^ CFU/100 mL) and spread-plated onto Mueller–Hinton (MH) agar (Neogen, Lansing, MI, USA) using sterile swabs dipped into the suspension. The antibiotic disks were dispensed onto the surface of the inoculated MH agar using an antibiotic disk dispenser and incubated at 37 °C for 24 h. Following incubation, the Petri dishes were examined for clear inhibition zones, which were measured (mm) and interpreted using the CLSI guidelines for *Staphylococcus* spp. and *Enterococcus* spp. as a surrogate (since resistance criteria is unavailable in the CLSI guidelines for *Listeria* spp.) [36].

Multiple antibiotic-resistant phenotypes (MARPs) were generated for bacterial isolates that were resistant to more than two antibiotics, using the method from Krumperman [15]. Resistance patterns, the number of antibiotics, and percentages were also recorded.

The mathematical expression from Krumperman [15] was used to calculate the multiple antibiotic resistance index (MARI) for each listerial isolate:

MAR index (MARI) *= n/m* (1)

where *n* is the number of antibiotics against which resistance was shown by a listeria isolate and *m* is the total number of antibiotics against which each listeria isolate was tested.

Three multiplex PCRs and one simplex PCR were used for the screening of 10 antibiotic resistance genes (ARGs) encoding Extended Spectrum Beta-Lactamases (ESBL), as previously described by Dallenne et al. [37]. Table 3 gives a summary of the group-specific primers and cycling conditions used for the detection of ESBL ARGs. Supplementary Material Table S1 gives a summary of all primers used for the screening of all the 31 target ARGs. The ARGs included *tet*A, *tet*B, *tet*C, *tet*D, *tet*K, *tet*M, *aac*C2, *aph*A1, *aph*A2, *aad*A, *str*A, *bla*_TEM_, *bla*Z, ampC, TEM, SHV, OXA1-like, GES, PER, VEB, ACC, FOX, MOX, DHA, CIT, EBC, *cml*A1, *cat*I, *cat*II, *Sul*I, and *sul*II. The PCR assays were either simplex, duplex, or multiplex. Briefly, 1 µL DNA was amplified in a 25 µL reaction mix made up of 12.5 μL master mix (Quick-Load BioLabs), 4.5 μL nuclease-free water, 5 μL DNA, 0.5 μL MgCl_2_, and buffer), a variable concentration of specific-group primers (Table 3 and Supplementary Material Table S1), and 1 U *Taq* polymerase (Sigma Aldrich, Johannesburg, South Africa). Amplification cycles were as presented in Table 3. Amplicons were electrophoresed at 100 V for 60 min using a 2% agarose gel spiked with 2 µL ethidium bromide. A 100 bp DNA ladder (New England Biolabs, Ipswich, MA, USA) was used as a size marker.

The data obtained were subjected to descriptive analysis using frequencies. The distribution of presumptive *Listeria* species in vegetable samples was represented via composite violin-box plots created using the ggpubr package (https://cloud.r-project.org/package=ggpubr, accessed on 7 June 2022) in R version 3.4.4 and compared by Kruskal–Wallis tests.

## 5. Conclusions

The presence of *L. monocytogenes* in fresh vegetables indicates a potential risk for consumers—especially the elderly, the immunocompromised, and pregnant women. The findings in this study indicate a high percentage of *L. monocytogenes* in the fresh vegetables studied and proves that fresh vegetables could be a reservoir of multidrug-resistant *L. monocytogenes* strains. Multidrug-resistant *L. monocytogenes* may serve as carriers of antibiotic resistance determinants that may be easily transferred to other bacteria in different environments, and possibly acquired by human pathogens through the ingestion of contaminated vegetables. To diminish the contamination of vegetables with multidrug-resistant microorganisms, it is imperative that vegetables are continuously monitored. It is also important to find ways to diminish the antibiotic selection pressure in order to reduce the dissemination of antibiotic-resistant foodborne pathogens.

## Figures and Tables

**Table 1:** MAR Phenotypes of *Listeria monocytogenes*.

No. of Antibiotics	Resistance Patterns	Frequency	MARI
3	E-PG-VA	3	0.18
4	E-PG-T-VA	1	0.25
4	E-PG-TS-VA	2	0.25
5	E-PG-T-TS-VA	6	0.25
5	AUG-E-PG-T-VA	3	0.31
5	CIP-E-PG-TS-VA	3	0.31
5	E-NI-PG-TS-VA	1	0.31
5	E-NI-PG-T-VA	1	0.31
6	CXM-E-PG-T-TS-VA	1	0.31
6	C-E-PG-T-TS-VA	1	0.31
6	E-NI-PG-T-TS-VA	6	0.31
6	AUG-E-PG-T-TS-VA	1	0.31
6	CIP-E-PG-T-TS-VA	1	0.31
6	KF-E-PG-T-TS-VA	4	0.38
6	AUG-CXM-E-PG-T-VA	2	0.38
6	CIP-E-MEM-PG-TS-VA	1	0.38
6	CIP-E-LEV-PG-TS-VA	2	0.38
6	CIP-E-NI-PG-TS-VA	1	0.38
6	AUG-CXM-KF-PG-T-VA	1	0.38
6	AUG-CXM-E-NI-PG-T	1	0.38
6	CXM-C-E-PG-T-TS	1	0.38
6	AUG-CXM-KF-CIP-MEM-PG	1	0.38
6	AUG-CXM-LEV-NI-PG-T	1	0.38
6	AUG-KF-E-PG-T-VA	1	0.38
6	AUG-C-E-PG-T-VA	1	0.38
6	AUG-CXM-KF-E-PG-T	1	0.38
6	AUG-CXM-KF-CIP-MEM-PG	1	0.38
6	AUG-CXM-LEV-NI-PG-T	1	0.38
6	AUG-KF-E-PG-T-VA	1	0.38
6	AUG-C-E-PG-T-VA	1	0.38
6	AUG-CXM-KF-E-PG-T	1	0.38
7	AUG-KF-CIP-E-PG-T-VA	1	0.44
7	AUG-CXM-KF-E-MEM-NI-PG	1	0.44
7	AUG-CIP-E-NI-PG-TS-VA	1	0.44
7	C-E-NI-PG-T-TS-VA	3	0.44
7	C-CIP-E-LEV-PG-TS-VA	1	0.44
7	CIP-E-LEV-NI-PG-TS-VA	2	0.44
7	AUG-KF-E-NI-PG-T-VA	1	0.44
7	AUG-CXM-E-PG-T-TS-VA	1	0.44
7	AUG-E-NI-PG-T-TS-VA	5	0.44
7	C-E-MEM-PG-T-TS-VA	1	0.44
7	CXM-E-NI-PG-T-TS-VA	2	0.44
7	AUG-CXM-KF-E-NI-PG-VA	1	0.44
8	C-CIP-E-NI-PG-T-TS-VA	1	0.5
8	CIP-E-MEM-NI-PG-T-TS-VA	1	0.5
8	E-K-MEM-NI-PG-T-TS-VA	2	0.5
8	KF-C-E-NI-PG-T-TS-VA	1	0.5
8	AUG-CXM-KF-E-NI-PG-T-VA	1	0.5
8	AUG-CXM-KF-E-PG-T-TS-VA	2	0.5
8	AUG-KF-E-NI-PG-T-TS-VA	1	0.5
8	AUG-E-MEM-NI-PG-T-TS-VA	1	0.5
8	AUG-CXM-E-NI-PG-T-TS-VA	1	0.5
8	AUG-CXM-KF-CIP-E-PG-TS-VA	1	0.5
8	AUG-CXM-KF-E-NI-PG-TS-VA	1	0.5
8	AUG-KF-E-LEV-PG-T-TS-VA	1	0.5
8	C-E-LEV-NI-PG-T-TS-VA	1	0.5
9	AUG-CXM-KF-E-MEM-NI-PG-T-VA	1	0.56
9	AUG-CXM-KF-E-NI-PG-T-TS-VA	1	0.56
9	AUG-CXM-E-MEM-NI-PG-T-TS-VA	1	0.56
9	AUG-CXM-E-GM-K-PG-T-TS-VA	1	0.56
9	AUG-CXM-KF-CIP-E-NI-PG-TS-VA	1	0.56
9	AUG-CXM-CIP-E-NI-PG-T-TS-VA	1	0.56
9	AUG-CXM-E-LEV-NI-PG-T-TS-VA	1	0.56
9	AUG-C-E-LEV-NI-PG-T-TS-VA	1	0.56
9	C-E-K-MEM-NI-PG-T-TS-VA	1	0.56
9	CXM-CIP-E-LEV-NI-PG-T-TS-VA	1	0.56
10	AUG-CXM-KF-E-LEV-NI-PG-T-TS-VA	1	0.63
10	AK-AUG-KF-E-MEM-NI-PG-T-TS-VA	1	0.63
10	AK-AUG-KF-CIP-E-LEV-PG-T-TS-VA	1	0.63
11	AUG-CXM-KF-C-E-K-NI-PG-T-TS-VA	6	0.69
13	AUG-CXM-KF-CIP-E-GM-K-LEV-NI-PG-T-TS-VA	1	0.81

**Table 2:** Frequency of antimicrobial resistance gene subtypes in *L. monocytogenes*.

Antimicrobial Family	Antimicrobial Agent	Antimicrobial Resistance Gene	No. of Positive Isolates	Percentage (%)
Tetracyclines	Tetracycline (*n* = 86)	*tet*A	51	59.3
		*tet*B	0	0
		*tet*C	37	43
		*tet*D	37	43
		*tet*K	1	1.2
		*tet*M	47	54.7
Aminoglycosides	Amikacin (*n* = 1) Gentamycin (*n* = 2) Kanamycin (*n* = 11)	*aac*C2	0	0
		*aph*A1	0	0
		*aph*A2	5	41.7
		*aad*A	4	33.3
		*str*A	0	0
Beta-lactams	Amoxicillin/Clavulanic Acid (*n* = 53) Penicillin G (*n* = 108)	*bla*_TEM_	19	17.6
		*bla*Z	0	0
		*amp*C	4	3.7
		TEM	83	76.9
		SHV	0	0
		OXA1-like	0	0
		GES	0	0
		PER	6	0
		VEB	0	0
Cephems	Meropenem (*n* = 50)	ACC	0	0
		FOX	16	32
		MOX	0	0
		DHA	3	6
		CIT	24	48
		EBC	0	0
Phenicols	Chloramphenicol (*n* = 20)	*cml*A1	7	35
		*cat*I	0	0
		*cat*II	1	5
Sulfanomides	Trimethoprim-Sulfamethoxazole (*n* = 86)	*Sul*I	86	100
		*sul*II	0	0

*n* = number of isolates tested.

**Table 3:** Group-specific primers and cycling conditions used for the detection of Extended Spectrum Beta-Lactamases (ESBL) antibiotic resistance genes (ARGs).

PCR Name	Primer	Primer Sequence	Amplicon Size (bp)	Cycling Conditions
Multiplex I TEM, SHV, and OXA-1-like	*bla*_TEM_, *bla*_SHV_, *bla*_OXA-1_	F: ATTTCCGTGTCGCCCTTATTC R: CGTTCATCCATAGTTGCCTGAC F: AGCCGCTTGAGCAAATTAAAC R: ATCCCGCAGATAAATCACCAC F: GGCACCAGATTCAACTTTCAAG R: GACCCCAAGTTTCCTGTAAGTG	800 713 564	Initial denaturation at 94 °C for 10 min; 30 cycles of 94 °C for 40 s, 60 °C for 40 s and 72 °C for 60 s; and a final elongation step at 72 °C for 7 min
Multiplex II FOX, CIT, and EBC	*bla*_FOX_ *bla*_CIT_ *bla*_EBC_	F: CTACAGTGCGGGTGGTTT R: CTATTTGCGGCCAGGTGA F: CGAAGAGGCAATGACCAGAC R: ACGGACAGGGTTAGGATAGY ^b^ F: CGGTAAAGCCGATGTTGCG R: AGCCTAACCCCTGATACA	162 538 683	Initial denaturation at 94 °C for 10 min; 30 cycles of 94 °C for 40 s, 60 °C for 40 s and 72 °C for 60 s; and a final elongation step at 72 °C for 7 min
Simplex CTX_M group 8/2	*bla*_CTX-M_	F: AACRCRCAGACGCTCTAC ^b^ R: TCGAGCCGGAASGTGTYAT ^b^	326	Initial denaturation at 94 °C for 10 min; 30 cycles of 94 °C for 40 s, 60 °C for 40 s and 72 °C for 60 s; and a final elongation step at 72 °C for 7 min
Multiplex III IMP, VIM, and KPC	*bla*_IMP_ *bla*_VIM_ *bla*_KPC_	F: TTGACACTCCATTTACDG ^b^ R: GATYGAGAATTAAGCCACYCT ^b^ F: GATGGTGTTTGGTCGCATA R: CGAATGCGCAGCACCAG F: CATTCAAGGGCTTTCTTGCTGC R: ACGACGGCATAGTCATTTGC	139 390 538	Initial denaturation at 94 °C for 10 min; 30 cycles of 94 °C for 40 s, 55 °C for 40 s and 72 °C for 60 s; and a final elongation step at 72 °C for 7 min
Multiplex IV GES and PER	*bla*_GES_ *Bla*_PER_	F: AGTCGGCTAGACCGGAAAG R: TTTGTCCGTGCTCAGGAT F: GCTCCGATAATGAAAGCGT R: TTCGGCTTGACTCGGCTGA	399 520	Initial denaturation at 94 °C for 10 min; 30 cycles of 94 °C for 40 s, 60 °C for 40 s and 72 °C for 60 s; and a final elongation step at 72 °C for 7 min

^b^ Y = T or C; R = A or G; S = G or C; D = A or G or T.

## Data Availability

The data presented in this study are available in this article and Supplementary Materials.

## Supplementary Materials

The following are available online at https://www.mdpi.com/article/10.3390/antibiotics11101353/s1, Table S1: Primers for profiling antimicrobial resistance determinants. References [38,39,40,41,42,43,44,45] are cited in the Supplementary Material.
