# Supplementary material for: Occurrence and Molecular Characterization of Multidrug-Resistant Vegetable-Borne Listeria monocytogenes Isolates
Source: Antibiotics (Basel). 2022 Oct 4;11(10):1353. doi: 10.3390/antibiotics11101353 (PMC9598391; doi:10.3390/antibiotics11101353)
Supplement: Supplementary file 1 [file antibiotics-11-01353-s001.zip › antibiotics-1785487-supplementary.pdf]

Table S1: Primers for profiling antimicrobial resistance determinants.

Antimicrobial family	Primer	Primer sequence	Amplicon size		Reference
**Sulphonamides**	*sul1*	F:TTCGGCATTCTGAATCTCAC<br>R:ATGATCTAACCCTCGGTCTC	822	Initial denaturation at 94°C for 5 min, followed by 1 min of denaturation at 94°C, 1 min of annealing at 55°C, 5 min of extension at 72°C for a total of 35 cycles and 5min of final extension at 72°C.	Maynard *et al.* (2004)
	Sul2	F: CGGCATCGTCAACATAACC<br>R: GTGTGCGGATGAAGTCAG	625	Initial denaturation for 5 min at 94°C, followed by 30 cycles of denaturation at 94°C for 30 s, annealing at 50°C for 30 s and extension at 72°C for 1.5 min and final extension at 72°C for 5 min.	Falbo *et al.* (1999)
**Beta-lactams**	*ampC*	F:TTCTATCAAMACTGGCARCC<br>R:CCYTTTTATGTACCCAYGA	550	Initial denaturation at 94°C for 4 min followed by 30 cycles: denaturation at 94°C for 45s, annealing at 60° C for 45s and extension at 72°C for 45s and final extension for 7 min at 72°C.	Velusamy *et al.* (2007)
	blaTEM	F:TTTCGTGTCGCCCTTATTCC<br>R:CCGGCTCCAGATTTATCAGC	690	Initial denaturation at 94°C for 5 min followed by 30 cycles of denaturation (94°C for 30 s), annealing (60°C for 30 s), extension (72°C for 90 s) and final extension at 72°C for 5 min.	Jannine *et al.* (2010)
	Blaz	F:ACT TCA ACA CCT GCT GCT TTC<br>R:TGACCACTTTTATCAGCAACC	490	Initial denaturation at 94°C for 5 min followed by 30 cycles of denaturation (94oC for 30 s), annealing	Baddour *et al.* (2007)

				(60°C for 30 s), extension (72°C for 90 s) and final extension at 72°C for 5 min.	
**Tetracyclines**	*tetA*	F:GCTACATCCTGCTTGCCTTC<br>R:CATAGATCGCCGTGAAGAGG	201	5 min initial denaturation at 94°C followed by 35 cycles of denaturation at 94°C for 1min, annealing at 55°C for 1min and extension at 72°C for 1.5 min and final extension at 72°C for 5 min.	Ng *et al.* (2001)
	tetB	F: TTGGTTAGGGGCAAGTTTTG<br>R:GTAATGGGCCAATAACACCG	359	5 min initial denaturation at 94°C followed by 35 cycles of denaturation at 94°C for 1min, annealing at 55°C for 1min and extension at 72°C for 1.5 min and final extension at 72°C for 5 min.	Ng *et al.* (2001)
	tetC	F:CTTGAGAGCCTTCAACCCAG<br>R:ATGGTCGTCATCTACCTGCC	418	5 min initial denaturation at 94°C followed by 35 cycles of denaturation at 94°C for 1min, annealing at 55°C for 1min and extension at 72°C for 1.5 min and final extension at 72°C for 5 min.	Ng *et al.* (2001)
	tetD	F:AAACCATTACGGCATTCTGC<br>R:GACCGGATACACCATCCATC	300	5 min initial denaturation at 94°C followed by 35 cycles of denaturation at 94°C for 1min, annealing at 55°C for 1min and extension at 72°C for 1.5 min and final extension at 72°C for 5 min.	Ng *et al.* (2001)
	tetK	F:GTAGCGACAATAGGTAATAGT<br>R:GTAGTGACAATAAACCTCCTA	460	5 min initial denaturation at 94°C followed by 35 cycles of denaturation at 94°C for 1min, annealing at 55°C for 1min and extension at 72°C for 1.5 min and final extension at 72°C for 5 min.	Strommenger *et al.* (2003)

	tetM	F:AGTGGAGCGATTACAGAA<br>R:CATATGTCCTGGCGTGTCTA	158	5 min initial denaturation at 94°C followed by 35 cycles of denaturation at 94°C for 1min, annealing at 55°C for 1min and extension at 72°C for 1.5 min and final extension at 72°C for 5 min.	Strommenger *et al.* (2003)
**Phenicols**	*cmlA1*	F:CACCAATCATGACCAAG<br>R:GGCATCACTCGGCATGGACATG	115	Initial denaturation at 94°C for 5 min followed by 30 cycles of denaturation at 94°C for 30 s, annealing at 50°C for 30 s, and extension at 72°C For 1.5 min and final extension at 72°C for 5 min.	Post and Hall (2009)
	catI	F:AGTTGCTCAATGTACCTATAACC<br>R:TTGTAATTCATTAAGCATTCTGCC	320	Initial denaturation for 5 min at 94°C, followed by 30 cycles of denaturation at 94°C for 30 s, annealing at 50°C for 30 s and 72°C for 1.5 min and final incubation at 72°C for 5 min.	Maynard *et al.* (2004)
	catII	F:ACACTTTGCCCTTTATCGTC<br>R:TGAAAGCCATCACATACTGC	543	5 min at 94°C, followed by 30 cycles of 94°C for 30 s, 50°C for 30 s and 72°C for 1.5 min and final incubation at 72°C for 5 min.	Maynard *et al.* (2004)
**Aminoglycosides**	*strA*	F:CTTGGTGATAACGGCAATTC<br>R:CCAATCGCAGATAGAAGGC	348	94°C for 4 min of initial denaturation, followed by 30 cycles of denaturation at 94°C for 45s, annealing for 45s at 50°C, extension at 72°C for 45s and final extension for 7min at 72°C.	Velusamy *et al.* (2007)
	aadA	F:GTGGATGGCGGCCTGAAGCC<br>R:AATGCCCAGTCGGCAGCG	525	Initial denaturation at 94°C for 4 min followed by 30 cycles of denaturation at 94°C for 45s, annealing at 50°C for 45 s and extension at 72°C for 45s and final extension for 7 min at 72°C	Velusamy *et al.* (2007)

aac(3)-IIa (aacC2)a	F:CGGAAGGCAATAACGGAG R:TCGAACAGGTAGCACTGAG	428	5 min initial denaturation at 94°C, followed by 30 cycles of denaturation at 94°C for 30 s, annealing at 50°C for 30 s and extension at 72°C for 1.5 min and final extension at 72°C for 5 min	Maynard *et al.* (2004)
aph(3)-Ia (aphA1)a	F:ATGGGCTCGCGATAATGTC R:CTCACCGAGGCAGTTCCAT	600	5 min initial denaturation at 94°C, followed by 30 cycles of denaturation at 94°C for 30 s, annealing at 50°C for 30 s and extension at 72°C for 1.5 min and final extension at 72°C for 5 min	Maynard et al. (2004)
aph(3)-IIa (aphA2)a	F:GAACAAGATGGATTGCACGC R:GCTCTTCAGCAATATCACGG	510	5 min initial denaturation at 94°C, followed by 30 cycles of denaturation at 94°C for 30 s, annealing at 50°C for 30 s and extension at 72°C for 1.5 min and final extension at 72°C for 5 min	Maynard et al. (2004)

PCR reaction mixture (25µl reaction volume):

**Component**	**Volume**
PCR Master Mix, 2X	12.5µl
Forward primer, 10µM	0.5µl
Reverse primer, 10µM	0. 5µl
DNA template	2.5µl
Nuclease-Free Water	9µl

Baddour, M.M., Abuelkheir, M.M.; Fatana, A.J. Comparison of mecA polymerase chain reaction with phenotypic methods for the detection of methicillin-resistant Staphylococcus aureus. *Curr. Microbiol.* 55, 2007, 473–479.

Falbo, V., Carattoli, A., Tosini, F., Pezzella, C., Dionisi, A.M.; Luzzi, I. Antibiotic resistance conferred by a conjugative plasmid and a class I integron in Vibrio cholerae O1 El Tor strains isolated in Albania and Italy. *Antimicrob. Agents Chemother.* 43, 1999, 693–696.

Jannine, K.B., Jeremy, L.P., Sashindran, A.; Ruth, M.H. Commensal Escherichia coli of healthy humans: a reservoir for antibiotic-resistance determinants. *J. Med. Microbiol.* 59, 2010, 1331–1339.

Maynard, C., Fairbrother, J.M., Bekal, S., Sanschagrin, F., Levesque, R.C., Brousseau, R., Masso,n L., Lariviere, S.; Harel, J. Antimicrobial resistance genes in enterotoxigenic Escherichia coli O149:K91 isolates obtained over a 23-year period from pigs. *Antimicrob. Agents Chemother.* 47, 2003, 3214–3221

Ng, L.K., Martin, I., Alfa, M.; Mulvey, M. Multiplex PCR for the detection of tetracycline resistant genes. *Mol. Cell. Probes* 15, 2001, 209–215

Post V. and Hall R.M. AbaR5, a large multiple-antibiotic resistance region found in Acinetobacter baumannii. *Antimicrob. Agents Chemother.* 53, 2009, 2667–2671.

Strommenger, B., Kettlitz, C., Werner, G.; Witte, W. Multiplex PCR assay for simultaneous detection of nine clinically relevant antibiotic resistance genes in Staphylococcus aureus. *J. Clin. Microbiol.* 41, 2003, 4089–4094

Velusamy, S., Barbara, E.G., Mark, J.L., Lien, T.N., Susan, I.H., Ynte, H.S., Stephen P.O. Phenotypic and genotypic antimicrobial resistance patterns of Escherichia coli isolated from dairy cows with mastitis, *Vet. Microbiol.* 124, 2007, 319–328.
